# Design Parameters for a Small-Gauge Fragmatome

**DOI:** 10.1167/tvst.8.4.21

**Published:** 2019-08-07

**Authors:** William J. Foster, Jijo Jizhou Wang

**Affiliations:** 1 Ophthalmic Research and Nanotechnology Group, Departments of Ophthalmology & Bioengineering, Temple University, Philadelphia, PA, USA; 2 Temple University School of Medicine, Philadelphia, PA, USA

**Keywords:** fragmatome, finite element, small gauge, lensectomy

## Abstract

**Purpose:**

Manufacturers of surgical instrumentation have increasingly sought to decrease the size of ophthalmic surgical instruments. We have used finite element modeling to model the stress and strain present in a fragmatome as a function of driving frequency and fragmatome dimensions.

**Methods:**

Finite element calculations using the COMSOL Multiphysics system v3.5 were used to elucidate the influence of wall thickness, length, and excitation frequency on a titanium fragmatome tube with outer diameters of 20, 23, 25, and 27 gauge.

**Results:**

By coupling structural mechanics, fluid mechanics, and acoustical physics, we were able to determine the eigenfrequencies (resonant frequencies) as well as parameters in which the von Mises stress in a fragmatome tube exceeds the yield strength, leading to destruction of the instrument.

**Conclusion:**

Solid fragmatomes have far fewer possible failure modes than fragmatomes with a standard wall thickness. Eigenfrequency analysis and finite element calculations can be critical in predicting potentially catastrophic designs in modern surgical instruments.

**Translational Relevance:**

Instruments developed for microsurgical applications cannot always simply be scaled down versions of conventional instruments. Such an approach can lead to potentially dangerous intraoperative failures, such as a fragmatome shattering inside the eye. Modern engineering techniques are increasingly necessary to investigate potential instrument failure mechanisms and to optimize device performance in a design in silico before in vivo testing.

## Introduction

Decrease in the size of an incision in surgery is associated with decreased pain,^1^–^3^ decreased rates of infection,^4^–^9^ better healing at the site of incision,^9^ and faster recovery^1^,^5^ in various surgical settings. Thus, the design, safety, and efficacy of smaller-diameter, modern surgical instruments are of growing interest.

In vitreoretinal surgery, with a decrease in incision size, the instrument gauge also must increase (i.e., the diameter of the instrument must decrease). In the case of retained lens material, a complication of cataract surgery, the retained lens fragments often are removed from the vitreous cavity by a fragmatome, a long tube that can apply ultrasonic energy to a lens fragment to emulsify it as well as to aspirate the resulting small lens particles. While modern vitrectors often can be used to remove soft nuclei and lens cortex, a fragmatome continues to be helpful in the management of hard nuclei. The advantages of a thinner fragmatome, such as a 23- and 25-gauge versus conventional 20-gauge fragmatome, have been well documented in terms of surgical efficiency,^5^ absence of sclerotomy sutures, which may then lead to reduced suture-induced astigmatism^10^ inflammation and pain postoperatively,^11^–^13^ as well as reduced intraocular inflammation.^14^ A retrospective study comparing the use of a 23-gauge fragmatome with and without a 20-gauge trocar reported that the tip of the fragmatome fractured and had to be surgically retrieved.^15^ This incident highlights a possible safety concern with smaller gauge instrumentation and, in particular, a smaller gauge fragmatome. We used finite element modeling to study the von Mises stress and strain present in a fragmatome as a function of frequency and fragmatome dimensions to predict potentially catastrophic designs. Finite element analysis is a computational tool that can be used for calculating forces, deformations, stresses, and strains at any point in a structure, such as a fragmatome.

## Methods

Finite element calculations using the COMSOL Multiphysics system v3.5 (Palo Alto, CA) were used to elucidate the influence of wall thickness, tube length, and excitation frequency on a titanium alloy fragmatome tube with outer diameters of 20, 23, 25, and 27 gauge. By coupling a linear elastic model of structural mechanics, fluid mechanics, and acoustical physics, we were able to determine the eigenfrequencies as well as parameters in which the internal von Mises stress (force/area) in the fragmatome exceeds the yield strength and, thus, according to the von Mises yield criterion,^16^ the fragmatome can break apart. The finite element calculations allowed for visualization of the von Mises stress and volumetric strain (change in volume/volume) of the fragmatome. The fragmatomes simulated were made of titanium.

Typical driving frequencies for phacoemulsification systems and fragmatomes vary from 35 to 55 kHz and, for some of our calculations, we chose a “typical” ultrasonic frequency of 45 kHz. For comparison with the calculations included in this study, the fragmatome used with Alcon's Constellation system operates at a frequency of 39.0 ± 1.9 kHz and has a length of 30.5 mm. Relevant engineering terms used in this study, with their definition, can be found in Appendix Table A1.

## Results

**Figure 1:**
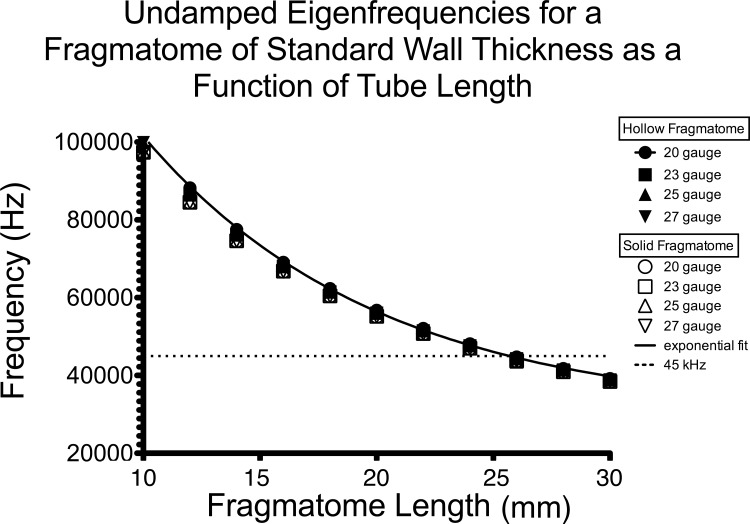
For a given fragmatome length, there is one undamped eigenfrequency (characteristic frequency) that does not depend upon gauge or wall thickness. The solid line represents the exponential one phase decay fit for the combined data points from 20-, 23-, 25-, and 27-gauge fragmatomes with a standard needle wall thickness. The constant, coefficient, and the goodness of fit for this exponential one phase decay fit are given in Table 1 under “All gauges combined.” As the fragmatome is made longer, the undamped eigenfrequency crosses 45 kHz (a frequency often used for clinical phacoemulsification) at approximately 25 mm length, leading to unexpected resonances in clinical instruments.

In our simulations, the undamped eigenfrequencies of a fragmatome are nearly independent of the gauge and wall thickness. Indeed, the relationship between eigenfrequency and length for 20-, 23-, 25-, 27-gauge fragmatomes of standard needle gauge wall thickness can be fit to an exponential one phase decay function with an *R*^2^ of 0.9996 (Fig. 1, Table 1) for all gauges. The exponential one phase decay fit for 20-, 23-, 25-, 27-gauge curves are statistically not different from each to other (Table 1). Likewise for a solid fragmatome, the relationship between eigenfrequency and tube length for 20-, 23-, 25-, and 27-gauge fragmatomes also can be fit with an exponential one phase decay function all with an *R*^2^ greater or equal to 0.999 (Table 2) and the exponential one phase decay fit for 20-, 23-, 25-, and 27-gauge are statistically not different from each other (Table 2). Comparing the decay constant *K* of an exponential one-phase decay fit of the solid fragmatomes to that of the hollow standard wall thickness fragmatomes, the decay constants are statistically not different between a solid and a hollow fragmatome for all gauges (Tables 1, 2). Based on analysis of variance (ANOVA) of the undamped eigenfrequencies at various fragmatome lengths, we did not find a statistically significant interaction between the groups analyzed, including different gauges, wall thicknesses, or the interaction between gauge and wall thickness (Table 3).

**Table 1:** Exponential One Phase Decay Fit: [y = (Y_0_ − Plateau) × e^−K × Length^ + Plateau] of Eigenfrequency vs. the Length of Standard Wall Thickness Fragmatome for 20-, 23-, 25-, and 27-Gauges.

**Gauge**	***Y*****_0_ (kHz)**	**Plateau (kHz)**	***K*** **(95% CI)**	***R*****^2^**
20	223	30.0	100 (93–106)	0.9996
23	218	29.8	99 (92–106)	0.9996
25	216	29.8	99 (92–106)	0.9996
27	217	29.8	100 (92–106)	0.9996
All gauges combined	218	29.8	100 (93–106)	0.999

**Table 2:** Exponential One Phase Decay Fit: [y = (Y_0_ − Plateau) × e^−K × Length^ + Plateau] of Eigenfrequency vs. the Length of Solid Fragmatome for 20-, 23-, 25-, and 27-Gauges.

**Gauge**	***Y*****_0_ (kHz)**	**Plateau (kHz)**	***K*** **(95% CI)**	***R*****^2^**
20	207	29.3	97 (91–103)	0.9996
23	207	29.3	97 (91–103)	0.9996
25	207	29.2	97 (91–103)	0.9996
27	207	29.3	97 (91–103)	0.9996
All gauges combined	207	29.3	97 (91–103)	0.9996

**Table 3:** Two-Way ANOVA of Eigenfrequency at Various Fragmatome Length as Indicated in Figures 1 and 2 Comparing Various Gauges (20-, 23-, 25-, 27-gauge) and Comparing Standard Wall Thickness vs. Solid Fragmatome

**Source of Variation**	**df**	***F***	***P* Value**	***F*****_crit_**
Wall thickness	1	0.096585	0.756775	3.960352
Gauge	3	0.00319	0.999749	2.718785
Interaction of gauge x wall thickness	3	0.003249	0.999742	2.718785

Alpha = 0.05. *P* < 0.05 indicates statistically significant variance between different groups.

**Figure 2:**
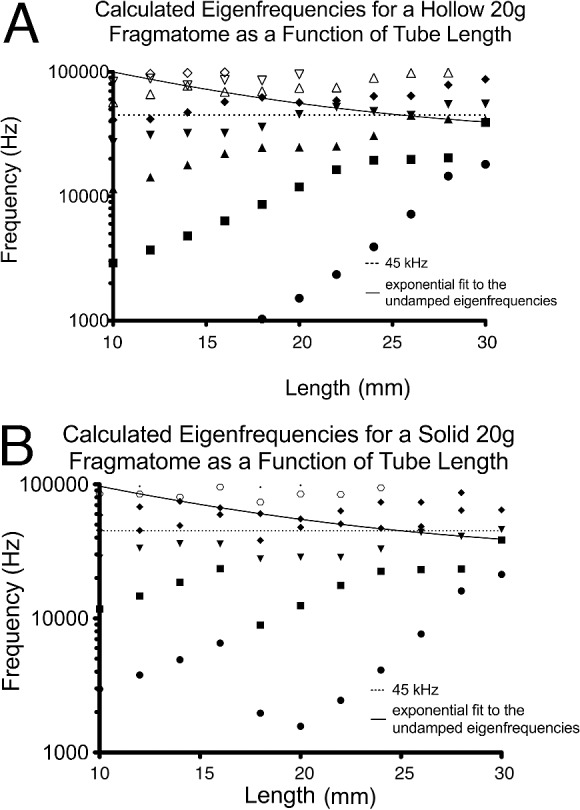
Calculated eigenfrequencies as a function tube length for (A) a hollow 20-gauge tube with a standard needle wall thickness in nine different families of eigenmodes and for (B) a solid 20-gauge tube in eight families of eigenmodes. The exponential one phase decay fit for the undamped eigenfrequencies of all gauges studied (20-, 23-, 25-, and 27-gauge) is drawn as a solid line. It is notable that the undamped eigenfrequencies at different fragmatome lengths belong to different families of eigenmodes. The normal excitation frequency used in most clinical instruments (45 kHz) is noted with a dashed line. These two lines are for reference and comparison.

As the length of the fragmatome increases, the undamped eigenfrequency decreases and eventually crosses 45 kHz, the driving frequency often used for clinical phacoemulsification, at a length of approximately 25 mm (Fig. 1). When the driving frequency, 45 kHz, is equal to the eigenfrequency, the system is considered to be at the resonant frequency of the fragmatome. At the resonant frequency, the maximum amplitude of displacement of the tip of the fragmatome is the greatest. Thus, as the fragmatome is made longer, the eigenfrequency of a given length of fragmatome approaches 45 kHz, leading to unexpected resonances in clinical instruments. Clearly, it also can be seen that the resonant frequency is less than 39 kHz for a fragmatome length of 30.5 mm, so Alcon's fragmatome is not driven at its resonant frequency.

Figure 2 shows eigenfrequencies of different eigenmodes, which describe the normal modes of vibration of a 20-gauge standard wall thickness (0.1524 mm) fragmatome (Fig. 2A) for the first to ninth eigenmodes of vibration and of a 20-gauge solid fragmatome (Fig. 2B) for the first to eighth eigenmodes of vibration. These eigenfrequencies are used in the finite element calculation to determine which eigenfrequency generates larger (here, we have produced figures for all eigenfrequencies >20 MPa) von Mises stress (Figs. 4A, 5A). ANOVA demonstrates that standard wall thickness and solid fragmatomes have statistically similar eigenfrequencies at various length for eigenmodes: first to third and fifth to eighth (Table 4). At the fourth eigenmode, the standard wall thickness and solid fragmatomes had statistically different eigenfrequencies at various lengths (Table 4).

**Table 4:** One-Way ANOVA of Eigenfrequency at Various Fragmatome Lengths, as Indicated in Figures 1 and 2, Comparing a Standard Wall Thickness vs. a Solid 20-Gauge Fragamatome in the First to Eighth Eigenmodes

**Eigenmode**	***F***	***P*** **Value**	***F*_crit_**
First	0.598846	0.44807	4.351244
Second	1.898533	0.183465	4.351244
Third	3.718177	0.068141	4.351244
Fourth	5.305936	0.032116	4.351244
Fifth	3.663355	0.070041	4.351244
Sixth	2.822174	0.108525	4.351244
Seventh	1.276445	0.27193	4.351244
Eighth	1.010908	0.33453	4.747225

Alpha = 0.05. *P* < 0.05 indicates statistically significant variance between the standard wall thickness vs. the solid fragmatome in that eigenmode.

**Figure 3:**
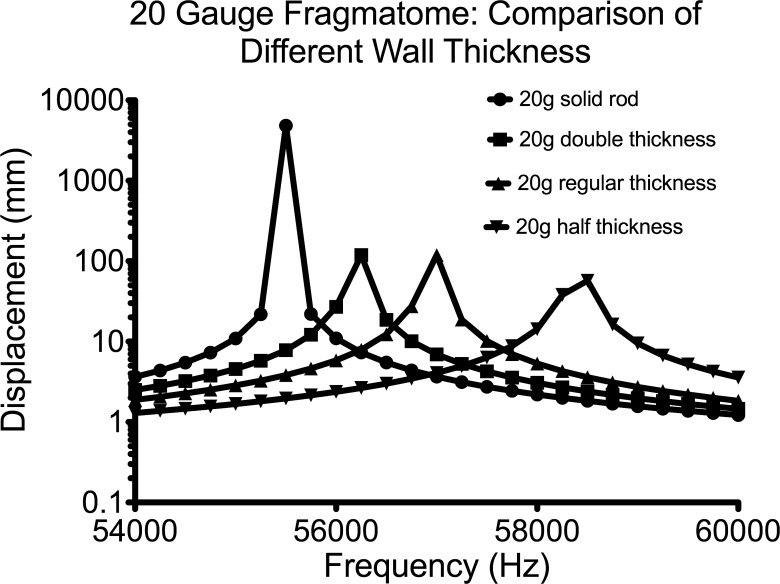
Plot of the displacement of the tip as a function of excitation frequency, demonstrating shifting amplitude (displacement from the tip of fragmatome) of the resonance with fragmatome tube construction. A 20-gauge tube has an outer diameter of 0.9081 mm. The wall thicknesses used in this simulation are: regular thickness = 0.1524 mm, half thickness = 0.0762 mm, double thickness = 0.3048 mm, solid is a solid rod.

Keeping the length (20 mm) and the gauge (20-gauge) of the fragmatome constant, the wall thickness can influence the maximum displacement of the tip of the fragmatome (Fig. 3). The displacement of the tip is 484.3 μm for a solid fragmatome at its resonance frequency. This displacement value is more than four times greater than that of hollow fragmatome of double the standard needle wall thickness, of the standard needle wall thickness, and of half the standard needle wall thickness (Fig. 3). The resonance frequencies are clustered around 56 kHz in concordance with the evidence that the undamped eigenfrequency is independent of wall thickness (Fig. 3).

**Figure 4:**
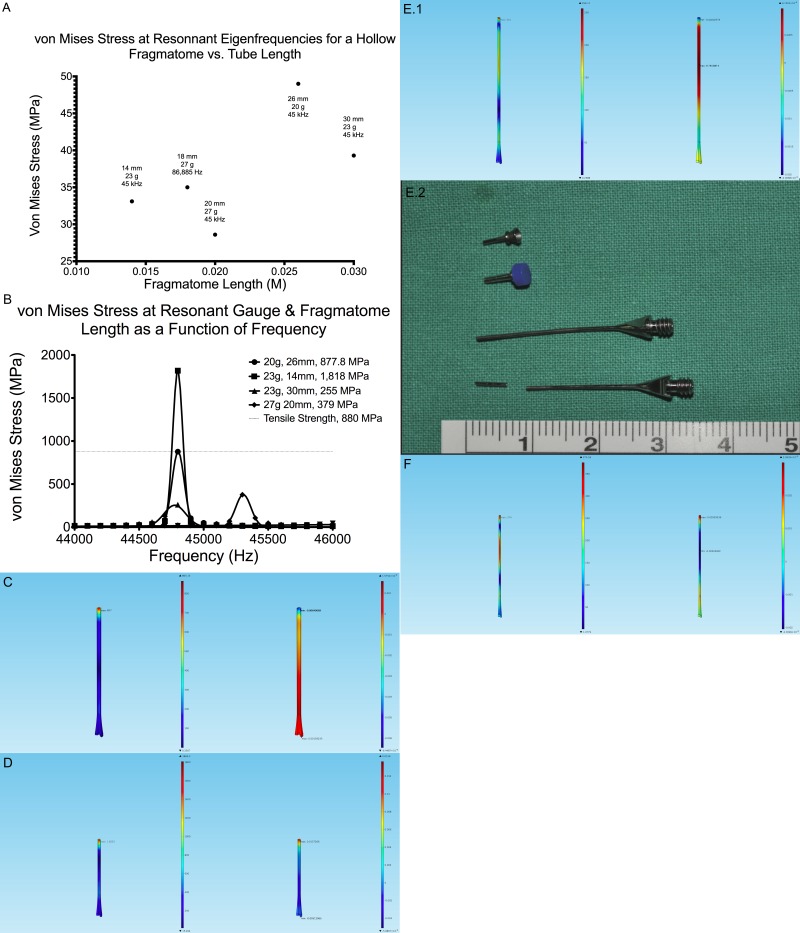
(A) Calculated elevated von Mises Stress for 20-, 23-, 25-, 27-gauge hollow fragmatomes at all predicted eigenfrequencies and 45 kHz. Note that at only a few combinations of fragmatome length and driving frequency are there resonances sufficiently strong to produce a von Mises stress above 40 MPa. (B) A plot of resonances near 45 kHz, as predicted in (A). (C) Von Mises stress (left) and volumetric strain (right) for a 26 mm long, 20-gauge hollow fragmatome at a frequency of 45 kHz. (D) Von Mises stress (left) and volumetric strain (right) for a 14 mm long, 23-gauge hollow fragmatome at a frequency of 45 kHz. (E.1) Von Mises stress (left) and volumetric strain (right) for a 30 mm long, 23-gauge hollow fragmatome at a frequency of 45 kHz. (E.2) Picture of an actual 23-gauge fragmatome that fractured during an operation,^16^ reproduced with permission of Kim et al.^15^ (F) Von Mises stress (left) and volumetric strain (right) for a 20 mm long, 27-gauge hollow fragmatome at a frequency of 45 kHz.

**Figure 5:**
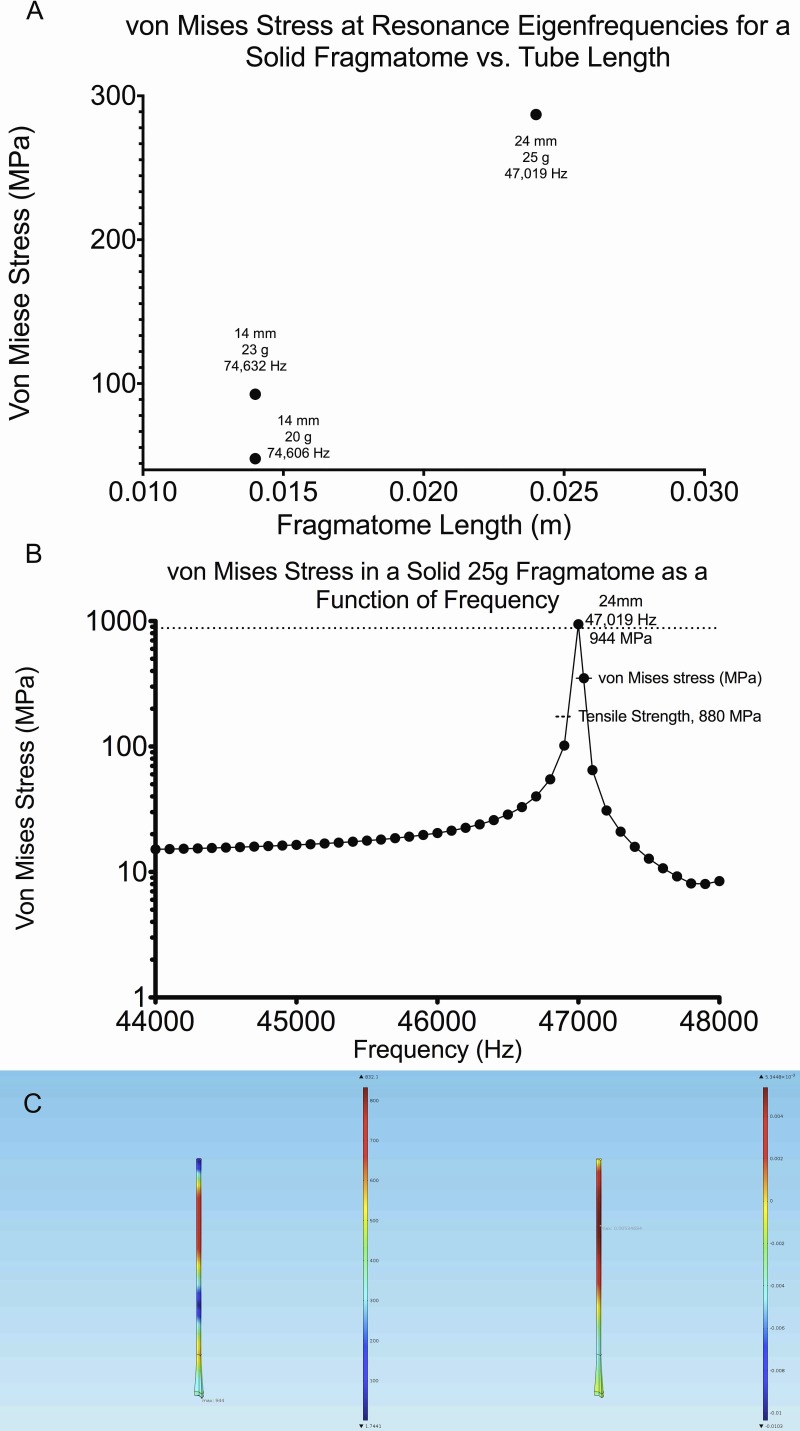
(A) Calculated elevated von Mises Stress for 20-, 23-, 25-, and 27-gauge solid fragmatomes at all predicted eigenfrequencies and 45 kHz. Note again that at only a few combinations of fragmatome length and driving frequency are there resonances sufficiently strong to produce a von Mises stress above 40 MPa. (B) The one resonance near 45 kHz, predicted in (A) is shown. (C) Von Mises stress (left) and volumetric strain (right) for a 24 mm long, 25-gauge solid fragmatome at a frequency of 47 kHz.

To predict potential failure, the von Mises stress was calculated for 20-, 23-, 25-, and 27-gauge hollow fragmatomes at all predicted eigenfrequencies and at 45 kHz at a series of lengths (Fig. 4A). Figure 4A demonstrates the frequencies that generate larger von Mises stress, at least 25 MPa, for a given fragmatome length. Larger von Mises stress was found for hollow fragmatomes, driven at 45 kHz, of dimensions: 20-gauge at 26 mm, 23-gauge at 14 and 30 mm, and 27-gauge at 20 mm (Fig. 4B).

At the tip of a 26 mm long, 20-gauge hollow fragmatome, driven at a frequency of 44.8 kHz, the volumetric strain calculation shows greatest compression at the tip and greatest extension at the base of the fragmatome (Fig. 4C). Driving such a fragmatome at a frequency of 44.8 kHz generates the maximal von Mises stress: 877.8 MPa, nearing the tensile strength of titanium, 880 MPa (Fig. 4B). This result predicts changes in shape (ductile failure^17^) or breakage during use (sudden failure^17^).

At the tip of a 14 mm long, 23-gauge hollow fragmatome, driven at a frequency of 45 kHz, the volumetric strain calculation shows greatest stretching at the tip of the fragmatome (Fig. 4D). Driving such a fragmatome at a frequency of 44.8 kHz generates the maximal von Mises stress: 1818 MPa, exceeding the tensile strength of titanium (880 MPa; Fig. 4B). This result predicts potential failure of a fragmatome with these properties when operated at approximately 45 kHz.

At the distal shaft of a 30 mm long, 23-gauge hollow fragmatome, driven at a frequency of 45 kHz, the volumetric strain calculation shows greatest stretching at the distal shaft of the fragmatome (Fig. 4E.1). As mentioned in the introduction, in a retrospective study, Kim et al.^15^ reported a case of a 23-gauge fragmatome fracturing at the distal shaft near the tip during the surgery; as can be seen in the photo of the broken fragmatome in that study, the length of fragmatome was approximately 30 mm (Fig. 4E.2).^15^ This catastrophic event provides supports for the validity of our finite element calculations.

At the distal shaft and tip of a 20 mm long, 27-gauge hollow fragmatome at a frequency of 45 kHz, the volumetric strain calculation shows greatest compression at the distal shaft and greatest stretching at the tip of the fragmatome (Fig. 4F).

To predict potential failure, von Mises Stress was calculated for 20-, 23-, 25-, and 27-gauge solid fragmatomes at all predicted eigenfrequencies and at 45 kHz at a series of lengths (Fig. 5A). Figure 5A demonstrates the frequencies that generate larger von Mises stress, at least 25 MPa, for a given fragmatome length.

Larger von Mises stress was found only in a 25-gauge, 24 mm long fragmatome, near 45 kHz (Fig. 5B). This fragmatome, when driven at a frequency of 47,019 Hz, develops a von Mises stress of 944 MPa, exceeding the tensile strength of titanium, 880 MPa (Fig. 5B). This result predicts that a fragmatome with these dimensions, when driven at 47,019 Hz, may undergo ductile failure or sudden failure during use. The maximal von Mises is present at the distal shaft of such a solid fragmatome and the volumetric strain shows greatest compression at the distal shaft of the fragmatome and greatest extension at the tip (Fig. 5C).

## Discussion

We find that the resonant frequencies at which a given fragmatome can vibrate vary strongly with the length of the fragmatome, and do not vary significantly with thickness or gauge. On the other hand, the thickness of the wall and the gauge strongly influence the internal stress and strain that develops in the device. Only by performing detailed computational modeling is it possible to determine sharp resonances in the response of a given fragmatome to a given ultrasonic frequency. It should be mentioned that simulations at different fragmatome length should be performed because, if the fragmatome is held firmly by the trocar in the sclera, the effective length of the fragmatome will be shortened and the eigenfrequencies of that shortened fragmatome are critical (analogous to a guitar string). In addition, if the driving force is not at an eigenfrequency, vibrations at an eigenfrequency will be induced as there is a coupling between the driving frequency and eigenfrequencies of the system.

There is a significant risk of intraoperative instrumentation failure, including having the end of the instrument move in an uncontrolled and large-amplitude manner or having a piece of a given fragmatome break off. These failure modes can be predicted using advanced computational modeling. We find, in our simulations, that these risks may be minimized by reducing the size of the fragmatome lumen.

Note that this analysis does not discuss failure due to fatigue, where the fragmatome is subject to repeated cyclical loading. Fatigue often results in the maximal stress that results in failure being much lower than the yield stress, considered here. In this way, our results are an upper limit on the stress needed to cause failure in a new fragmatome and the actual risk of failure is is often computed using statistical considerations.

Eigenfrequency analysis and finite element calculations can be critical in optimal instrument design and in predicting potentially catastrophic designs in modern surgical instruments. For example, the calculated stress and strain for a 30 mm long, 23-gauge hollow fragmatome at a frequency of 45 kHz demonstrates a maximal von Mises stress that exceeds tensile strength of titanium, (880 MPa) at the distal shaft of the fragmatome. Our calculations, thus, predict the fracture of such a fragmatome, as has been noted in a published report of fracture of a 23-gauge fragmatome.^15^ As the trend toward miniaturization of surgical instrumentation proceeds, such modern engineering analysis will become increasingly crucial. In addition, such techniques may allow optimization of a device under design.

## Summary

It is possible to use finite element calculations to predict the complex motion of a surgical device, such as a fragmatome, as the thickness of the wall of the fragmatome tube or the length of the fragmatome tube is changed. We found that changing the thickness of the wall of the fragmatome did not change the primary frequency that the device vibrated at and the length of the fragmatome changed the primary frequency in a predictable manner. In addition, there were very narrow ranges of frequencies over which the vibration of the device suddenly became large (a resonance), sometimes large enough to break apart a titanium device. In fact, we were able to predict the failure of a 30 mm fragmatome, which occurred clinically and was previously reported. Such resonances require a calculation and cannot be easily predicted. Thus, instruments developed for microsurgical applications cannot always be simply scaled down versions of conventional instruments. Such an approach can lead to potentially dangerous intraoperative failures, such as a fragmatome shattering inside the eye. Modern engineering techniques are increasingly necessary to investigate potential instrument failure mechanisms and to optimize device performance in a design in silico before in vivo testing.

## Appendix

**Table A1:** Table of Engineering Terms Used in this Study

**Term**	**Definition**
Finite element calculation	A form of performing calculations using a computer in which the system being modeled (for example, a fragmatome needle in water) is broken up into a fine grid where the motion of each point in the grid is computed using the equations that describe structural mechanics, fluid mechanics, and also acoustical physics at the same time (i.e., coupled). The motion of the entire system is calculated over a short period of time and, in this way, the motion of the entire system is predicted. Discussion of technical details (such as the size of the grid or the time step) are carefully determined but are beyond the scope of this study.
Eigenfrequency	The characteristic or resonant frequency at which an item will 'ring' or vibrate. Note that the name is from German, where eigen means inherent or characteristic. The term eigenfrequency is, according to the Merriam-Webster dictionary,^18^ “One of the frequencies with which a given oscillatory system is capable of vibrating.” The motion of a given system, such as a fragmatome, can be described by a combination of its eigenfrequencies in the same way as a musical sound can be described by the frequencies that make up that sound. Consider that a stick that can oscillate at a given frequency, such that the entire stick moves except the ends; or it can oscillate at a frequency such that there is a point in the center of the stick that also does not move ... Each such frequency at which the stick can oscillate is an eigenfrequency and the stick, when struck, will oscillate at a combination of these eigenfrequencies. Another way to think about this is the equalizer on a stereo system. There often is a screen that displays frequency (along the *x*-axis) versus power (along the *y*-axis). This shows that sound (or any function) can be broken down into a number of frequencies. Critically, many frequencies will be quickly damped out and only certain frequencies, the eigenfrequencies, will persist and these frequencies determine the behavior of the system.
Undamped eigenfrequencies	The eigenfrequencies that are present and would be present if there were no damping from the surrounding fluid.
Damped eigenfrequency	It is possible to calculate how rapidly a particular vibration (eigenfrequency) will lose energy to the surrounding fluid, and, thus, will have lower amplitude vibration because of how the fragmatome moves. We use this term to denote eigenfrequencies that lose energy.
